# Characterization of the complete chloroplast genome of *Syzygium nervosum*

**DOI:** 10.1080/23802359.2021.1894999

**Published:** 2021-03-18

**Authors:** Pan Li, Weiyue Guo, Kang Lei, Lusha Ji

**Affiliations:** School of Pharmacy, Liaocheng University, Liaocheng City, People’s Republic of China

**Keywords:** *Syzygium nervosum*, Myrtaceae, complete chloroplast genome

## Abstract

*Syzygium nervosum A.Cunn. ex DC* (also named as *Cleistocalyx operculatus*) is a member of genus *Syzygium* mainly distributed in China, Vietnam and some other tropical countries and belongs the family of *Myrtaceae*. *Syzygium nervosum* is a popular medicinal plant, some species of genus *Syzygium* have been used in folk medicine. In this study, we sequenced the sample of *Syzygium nervosum* and determined its complete chloroplast genome. The complete chloroplast genome of *Syzygium nervosum* is 158,979 bp in length, and contained a large single copy (LSC) with 88,099 bp in length, a small single copy (SSC) with 18,756 bp in length and two inverted repeat (IR) regions of 26,062 bp each. It includes 85 protein coding genes, 8 rRNA and 37 tRNA, and 37% overall GC content. Each of *trn*K-UUU*, rps*16, *trn*G-UCC*, atp*F*, rpo*C1*, trn*L-UAA*, trn*V-UAC*, pet*B*, pet*D*, rpl*16*, rpl*2*, ndh*B*, trn*I-GAU, *trn*A-UGC and *ndh*A genes contain an intron, the gene *clp*P and *ycf*3 contain 2 introns. The phylogenetic position shows that *Syzygium nervosum* has the closest relationship with *Syzygium aromaticum*.

*Syzygium nervosum A.Cunn. ex DC* (also named as *Cleistocalyx operculatus*) is a member of genus *Syzygium* mainly distributed in China, Vietnam and some other tropical countries and belongs the family of *Myrtaceae* (Tran et al. 2019). *S. nervosum* is a kind of popular medicinal plant, the buds of *S. nervosum* have been used in various beverages in Southern China since ancient times (Woo et al. 2002; Mai and Chuyen 2007; Dung et al. 2009). *S. nervosum* also is the important raw martial of essential oil that had excellent anti-inflammatory activities both in vitro and in vivo (Dung et al. 2009). People have learnt *S. nervosum* for hundreds of years and constantly discovered new function of *S. nervosum* for human ’s life. However, the study about complete chloroplast genome of *Syzygium* was very deficient especially *S. nervosum*, and there was no information about complete chloroplast genome of *S. nervosum* in database of NCBI. So in this study we sequenced the sample of *S. nervosum* and determined its complete chloroplast genome.

The sample of *Syzygium nervosum* was collected from South China Botanical Garden, Tianhe District, Guangzhou, Guangdong Province (N113°22′34″, E23°11′32″), and the fresh leaves had been used to extract total genomic DNA based CTAB method (Doyle and Doyle 1987) and construct the libraries with an average length of 350 bp using the NexteraXT DNA Library Preparation Kit (Illumina, San Diego, CA). Then the libraries were sequenced on Illumina Novaseq 6000 platform, 3.05 Gb Illumina raw sequence reads were edited using the NGS QC Tool Kit v2.3.3. High-quality reads were assembled into contigs using the de novo assembler SPAdes 3.11.0 software (Bankevich et al. 2012) and annotated by Plann software (Huang and Cronk 2015). The complete sequence and annotation results were submitted to GenBank, under the accession number (MW054699) and the sample was stored at Laboratory of Molecular Biology, Liaocheng University, Liaocheng. (Voucher specimen: CO20200701LP) (Lusha Ji, email: jilusha2020@163.com).

The complete chloroplast genome of *Syzygium nervosum* is 158,979 bp in length, and contains a large single copy (LSC) with 88,099 bp in length, a small single copy (SSC) with 18,756 bp in length and two inverted repeat (IR) regions of 26,062 bp each. There are 130 genes, which includes 85 protein coding genes, 8 rRNA and 37 tRNA, and 37% overall GC content. Each of *trn*K-UUU, *rps*16, *trn*G-UCC, *atp*F, *rpo*C1, *trn*L-UAA, *trn*V-UAC, *pet*B, *pet*D, *rpl*16, *rpl*2, *ndh*B, *trn*I-GAU, *trn*A-UGC and *ndh*A genes contain an intron, the gene *clp*P and *ycf*3 contain 2 introns.

To confirm the phylogenetic position and understand the relationship of *Syzygium nervosum* within *Myrtaceae*. The complete chloroplast genome of 13 species in family *Myrtaceae* were collected and aligned with *Syzygium nervosum* by MAFFT7.037 (Katoh and Standley 2013). Subsequently, the phylogenetic tree was constructed by IQTREE v1.6 (Nguyen et al. 2015; Hoang et al. 2018) with 1000 bootstraps replicates using Best-fit model. By using *Canavalia rosea* (LC554221) as out group we got the final ML tree, then the Figure 1 shows that *Syzygium nervosum* has the closest relationship with *Syzygium aromaticum* (MN746306.1).

**Figure 1.**
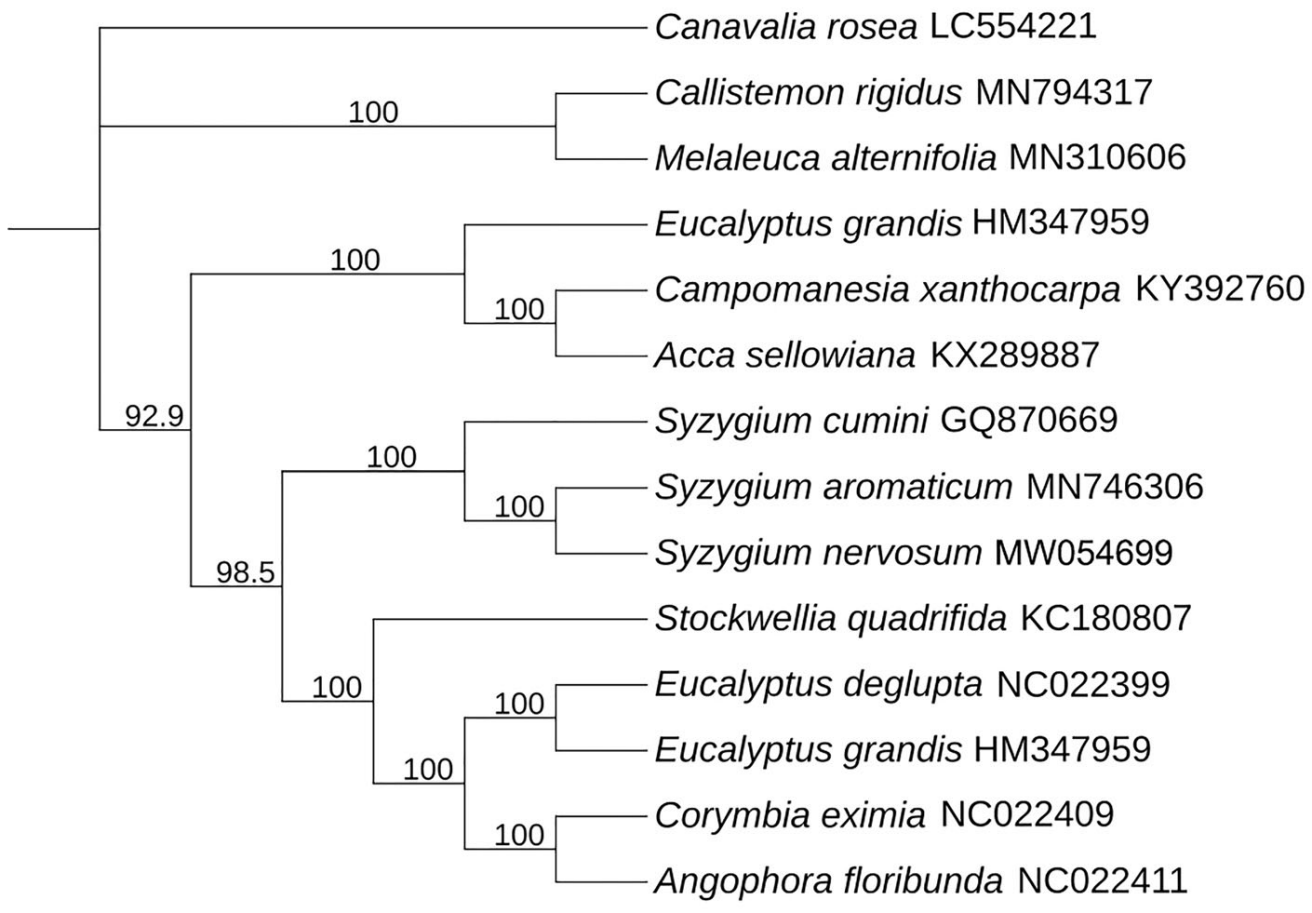
Maximum-likelihood phylogenetic tree for *Syzygium nervosum* based on 14 species complete chloroplast genomes in family *Myrtaceae*.

## Data Availability

The chloroplast genome sequence data that support the findings of this study are openly available in GenBank of NCBI at (https://www.ncbi.nlm.nih.gov/) under the accession no. MW054699. The associated BioProject, SRA, and Bio-Sample numbers are PRJNA693034, SRR13479128, and SRS8071277, respectively.
